# Measuring antimicrobial prescribing quality in outpatient parenteral antimicrobial therapy (OPAT) services: development and evaluation of a dedicated national antimicrobial prescribing survey

**DOI:** 10.1093/jacamr/dlaa058

**Published:** 2020-08-06

**Authors:** N Deborah Friedman, Seok M Lim, Rodney James, Robyn Ingram, Mary O’Reilly, James G D Pollard, Sonia Koning, Catherine George, Arjun Rajkhowa, Douglas F Johnson, Kirsty L Buising

**Affiliations:** 1 National Centre for Antimicrobial Stewardship, Melbourne, VIC, Australia; 2 Barwon Health, Geelong, VIC, Australia; 3 Department of General Medicine, Royal Melbourne Hospital, Melbourne, VIC, Australia; 4 Eastern Health, Melbourne, VIC, Australia; 5 Pharmacy Department, Royal Melbourne Hospital, Melbourne, VIC, Australia; 6 Department of Infectious Diseases, Royal Melbourne Hospital, Melbourne, VIC, Australia; 7 Cabrini Health, VIC, Australia

## Abstract

**Background:**

Antimicrobial stewardship programmes are important in driving safety and quality of antimicrobial prescribing. The National Antimicrobial Prescribing Survey (NAPS) is a point-prevalence audit of inpatient antimicrobial prescribing in Australian hospitals.

**Objectives:**

To design and adapt the NAPS tool for use in the outpatient parenteral antimicrobial therapy (OPAT) and hospital-in-the-home (HITH) setting.

**Methods:**

An inter-disciplinary working group with expertise in OPAT and HITH services was established to adapt the NAPS template for use in the OPAT setting—called HITH-NAPS. This was initially trialled in 5 HITH services, subsequently adapted following participant feedback, then offered nationally to 50 services in 2017.

**Results:**

There were 1154 prescriptions for 715 patients audited via the HITH-NAPS. The most common antimicrobials prescribed were cefazolin (22%), flucloxacillin (12%), piperacillin/tazobactam (10%) and ceftriaxone (10%). The most common infections treated were cellulitis (30%) and respiratory tract infections (14%). Eighty-seven percent of prescriptions were assessed as appropriate, 11% inappropriate and 2% not assessable. Prolonged durations of antimicrobials and unnecessarily broad-spectrum antibiotics were used in 9% of prescriptions.

**Conclusions:**

The HITH-NAPS pilot project revealed that auditing of this type is feasible in HITH. It showed that antibiotic use in these HITH services was generally appropriate, but there are some areas for improvement. A national OPAT/HITH-NAPS can facilitate benchmarking between services, identify potentially inappropriate prescribing and help guide quality improvement.

## Introduction

WHO has described antimicrobial resistance (AMR) as one of the main threats to human health.^1^ MDR infections lead to worse morbidity and mortality outcomes and are associated with substantial treatment costs.^2^ Globally, optimizing the management of infections in hospitalized people is a key strategy to reduce AMR development.^3^^,^^4^ ‘Antimicrobial Stewardship in Australian Hospitals’, published by the Australian Commission on Safety and Quality in Health Care in 2011, offers a framework for improving the treatment and prevention of infections through more judicious use of antimicrobials, utilization of antimicrobial stewardship (AMS) programmes, clinical guideline development and clinical audit in hospitals.^5^^,^^6^

The National Antimicrobial Prescribing Survey (NAPS) was developed in 2011 by a team comprising infectious diseases physicians, clinical microbiologists and pharmacists at the National Centre for Antimicrobial Stewardship (NCAS), Melbourne, Australia, with the aim of identifying areas of inappropriate antimicrobial use to directly inform ongoing AMS activities and drive improvement in clinical practice.^7^ NAPS uniquely focuses on the quality of antimicrobial prescribing by measuring the appropriateness of individual prescriptions of antimicrobials—taking into account drug choice, dose, duration for a nominated indication and compliance with published guidelines—and is an important tool in meeting key objectives of Australia’s First National Antimicrobial Resistance Strategy (2015–19).^3^^,^^8^ NAPS has been widely adopted in Australian hospitals, and has also been adapted to suit other contexts in which antimicrobials are used, such as residential aged care facilities.

Outpatient parenteral antimicrobial therapy (OPAT), first developed in the 1970s,^9^ enables patients to be given IV antibiotics in the community rather than in hospital. In Australia, OPAT is mostly delivered via hospital-in-the-home (HITH) services, an acute bed substitution programme for patients requiring treatment traditionally delivered in a ward bed.^10^^,^^11^ OPAT and HITH programmes can aid in minimizing risks associated with prolonged hospitalization and in optimizing appropriate use of hospital ward beds.^12^Hospital-based care is costly, and associated with high incidences of complications such as delirium, functional decline, hospital-acquired infections and medication errors, particularly amongst frail, older individuals.^13^

For both OPAT and HITH services, antibiotic use is contingent upon the ability to deliver this safely in the community, and this requirement can lead to broad-spectrum agents being chosen in preference to potentially more efficacious agents that are unable to be administered in the home setting.^14^^,^^15^ Another potential avenue for inappropriate antimicrobial usage in OPAT is overuse of IV systemic agents because the incentive to switch to oral medication to enable hospital discharge is reduced. As such, OPAT practice guidelines include recommendations to ‘monitor for complications of treatment or the program’ and also stress the importance of close involvement by the local AMS team in the OPAT program.^16–19^ The emergence of OPAT has occurred in parallel with an increased focus on promoting the prudent and rational use of antimicrobials through AMS programmes,^1^ and OPAT antimicrobial plans should follow stewardship principles similar to those developed for inpatients.^20^

The aim of this study was to modify the Hospital NAPS for use in the OPAT setting to enable collection of information about the prescribing of antimicrobial agents, including information about indications and duration, and to perform a pilot study of this prescribing audit among HITH services in Australia. Results from this audit would allow benchmarking between HITH services and auditing of appropriateness of prescribing, ultimately to provide data to identify any problems and guide improvement in clinical practice.

## Methods

### Working group development

In March 2016, NCAS established an inter-disciplinary working group with expertise in the HITH setting, comprising infectious diseases physicians and AMS pharmacists to determine the optimal methods for a national HITH antimicrobial audit.

The working group created a survey for HITH services and modified the existing Hospital NAPS data fields to suit the HITH setting, thus creating the HITH-NAPS (Appendix S1, available as Supplementary data at *JAC-AMR* Online). The HITH-NAPS was initially piloted in 2016 in five HITH services in Victoria to address usability and feasibility concerns. Subsequently, the pilot was extended to all Australian HITH services from April to July 2017. Fifty HITH-NAPS surveys were posted to existing Hospital NAPS registrants and members of the Australasian HITH society who expressed interest following a period of publicity about the study.

### Data collection

The HITH-NAPS was piloted utilizing a paper-based audit form and Microsoft Excel™ datafiles. Data were collected either prospectively or retrospectively by local auditors including physicians, pharmacists or HITH nurses, and each HITH service was asked to include at least 30 antimicrobial prescriptions. Auditors were trained using an online module (already used for Hospital NAPS) and support was provided through phone support by dedicated AMS staff. Data were collected on consecutive antimicrobial prescriptions, including the dose, frequency and method of administration, indication and duration of therapy via HITH. Compliance with prescribing guidelines was judged according to recommendations in the current version of ‘Australian Therapeutic Guidelines: Antibiotic’^21^ or any locally endorsed guidelines. Antimicrobials that were considered to be prescribed in response to microbiology results were classified as directed therapy. Assessments of appropriateness of the prescription were determined using the Hospital NAPS Appropriateness Definitions (Appendix S2) and were defined as appropriate (included optimal or adequate), inappropriate (included suboptimal or inadequate) or not assessable. Where a prescription was assessed as inappropriate the auditor recorded the reason why this assessment was made.

### Data analysis

HITH-NAPS data were analysed utilizing SPSS. Descriptive statistics were generated to describe the characteristics of the sample, and antimicrobial data were described using numbers and percentages. A sub-analysis of parenteral antimicrobials utilized for respiratory tract infections (RTIs) was conducted with exclusion of oral and inhaled antimicrobial agents.

### Ethics

Ethics approval for the project was obtained through the Melbourne Health Human Research Ethics Committee (project number QA2013066).

## Results

A total of 23 HITH services throughout the Australian states of Victoria, New South Wales, Queensland and Tasmania participated in the 17 week HITH-NAPS pilot. Thirteen regional services were represented in this study.

Patients were referred to HITH services from emergency departments (40%), general and acute care medical units (28%), orthopaedic units (19%) and infectious diseases departments (13%). In total, 1154 prescriptions for 715 patients (63% male) were included. Patients ranged in age from 1 month to 101 years and the median age was 58 years.

**Figure 1.**
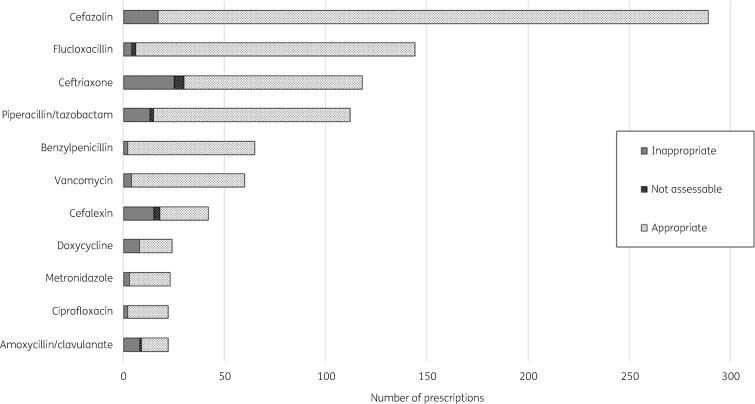
Most commonly prescribed antimicrobials in the HITH-NAPS audit.

**Figure 2.**
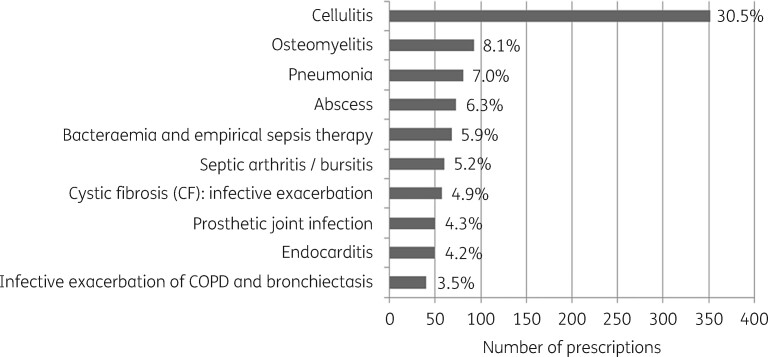
Most common indications for prescribing an antimicrobial in the HITH-NAPS audit.

The most common antimicrobials prescribed were cefazolin (22%) and flucloxacillin (12%) (Figure 1). The most common indications for antimicrobials were cellulitis (30%) and osteomyelitis (8%), while a composite of all RTIs accounted for 14% (Figure 2). The median duration of parenteral therapy for cellulitis was 4 days; however, duration ranged overall from 1 to 44 days for this indication.

**Figure 3.**
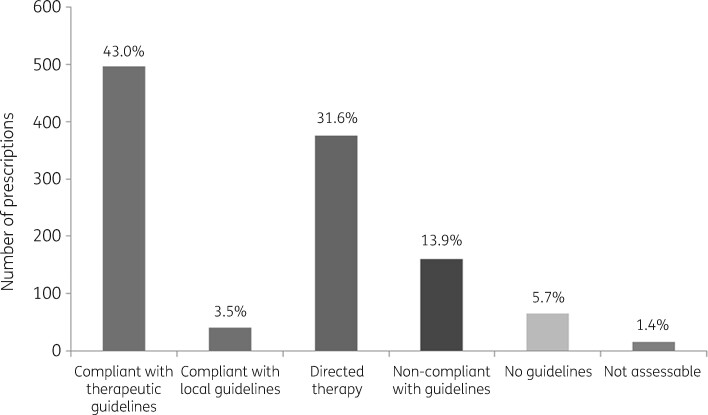
Antimicrobial prescriptions’ compliance with national and local guidelines in the HITH-NAPS audit.

Further analysis of specific indications for antimicrobial therapy and the specific agents prescribed was undertaken. Overall, 46.5% of prescriptions were compliant with Australian guidelines, with 3.5% of these utilizing locally relevant guidelines. An additional 31.6% of prescriptions were considered directed therapy targeting a specific causative organism (Figure 3). Utilizing the NAPS appropriateness assessment guide, auditors assessed 87% of prescriptions as appropriate, 11% as inappropriate and 2% unable to be assessed. Cefazolin (94.1%), flucloxacillin (95.8%) and vancomycin (93.3%) were antimicrobials most commonly assessed to be appropriately utilized. Ceftriaxone, particularly in the context of pneumonia treatment, was the antimicrobial most frequently assessed as inappropriate (*n* = 25). Of 118 ceftriaxone prescriptions, 26 (22%) were assessed as being unnecessarily broad-spectrum for the condition being treated and 15 (12.7%) as excessively prolonged in terms of the duration of treatment. Overall, antimicrobial therapy duration was suboptimal in 9% of cases, and antibiotic spectrum was considered too broad in 9% of prescriptions.

Sub-analysis of parenteral antimicrobial prescribing for RTIs indicated that there were 127 parenteral antimicrobial prescriptions among 88 patients, most commonly for pneumonia (46%), exacerbations of cystic fibrosis (31%) and infective exacerbations of bronchiectasis (15%). Forty-seven out of 59 antimicrobial prescriptions (80%) for pneumonia were non-compliant with local guidelines. Common parenteral antimicrobials prescribed for RTIs were ceftriaxone (39%), piperacillin/tazobactam (20%), tobramycin (9%) and ceftazidime (9%) (Table 1). Among all prescriptions for RTIs, there were an additional 43 prescriptions for oral antibiotics, and 9 prescriptions for inhaled antibiotics that were excluded from this analysis of appropriateness of prescribing of IV antibiotics by OPAT.

**Table 1.** Most commonly prescribed parenteral antimicrobials for RTIs within the HITH-NAPS audit

Parenteral antimicrobial for RTIs	Prescriptions, *n* (%)
Ceftriaxone	49 (39)
Piperacillin/tazobactam	25 (20)
Tobramycin	12 (9)
Ceftazidime	11 (9)
Azithromycin	8 (6)
Meropenem	7 (5)
Benzylpenicillin	5 (4)
Cefazolin	3 (2)
Flucloxacillin	2 (2)
Metronidazole	2 (2)
Cefepime	2 (2)
Gentamicin	1 (1)

Analysis of the prescriptions for broad-spectrum agents indicated that piperacillin/tazobactam was the most frequently prescribed broad-spectrum antimicrobial in Australian HITH services. There were 21 documented indications in HITH-NAPS for the use of piperacillin/tazobactam, with 33% of piperacillin/tazobactam prescriptions being judged as compliant with guidelines. Some of the indications assessed as inappropriate included cellulitis, pyelonephritis and infective exacerbations of COPD without pathogens identified (Table 2). Carbapenems were prescribed for indications such as prostatitis, osteomyelitis, pyelonephritis and intra-abdominal abscesses; the majority of carbapenem prescriptions were for directed therapy for known pathogens.

**Table 2.** Indications for the prescribing of piperacillin/tazobactam in the HITH-NAPS audit

Indications for piperacillin/tazobactam	Prescriptions, *n* (%)
Osteomyelitis	27 (26)
Bronchiectasis	12 (11)
Diabetic infection	10 (10)
Cystic fibrosis: infective exacerbation	9 (9)
Cellulitis	9 (9)
Prosthetic joint infection	5 (5)
Septic arthritis	4 (4)
Sepsis	4 (4)
Abscess	4 (4)
Surgical wound infection	3 (3)
Pyelonephritis	3 (3)
Cholangitis	3 (3)
Skin ulcers	2 (2)
Prostatitis	2 (2)
Intravascular prosthesis infection	2 (2)
Bites and clenched fist wound infections	2 (2)
Community acquired pneumonia	1 (1)
Otitis externa	1 (1)
Endocarditis	1 (1)
COPD: infective exacerbation	1 (1)

Appropriateness and compliance with antimicrobial therapy guidelines in terms of antimicrobial utilization tended to correlate with the occurrence of review by an infectious diseases physician either prior to or during patient management under HITH. This was particularly evident where a pathogen had been isolated on culture (Table 3).

**Table 3.** Correlation of appropriateness of antimicrobial prescriptions with occurrence of infectious diseases consultations within the HITH-NAPS audit

	With infectious diseases consultation, *n* (%)	Without infectious diseases consultation, *n* (%)	*P* value
Optimal and adequate antimicrobial prescribing	485/514 (94.4)	515/637 (80.8)	<0.0001
Guideline-compliant directed therapy	281/514 (55)	95/637 (15)	<0.0001
Non-guideline-compliant prescribing	33/514 (6.4)	95/637 (15)	<0.0001

## Discussion

The HITH-NAPS pilot was undertaken by 23 Australian HITH services, several of which were outside of metropolitan areas. While all hospitals in Australia do have access to AMS programmes, resources are likely to be scarcer in smaller centres. The utilization of HITH and OPAT is known to be expanding across a variety of healthcare settings and organizations, including through the expansion of non-infection-specialist-led services.^15^^,^^19^ Obtaining antimicrobial prescribing data from a variety of services, including those in non-metropolitan settings and those without infectious diseases input, assists in strengthening our understanding of how prescribing may be influenced by resources and geographical location.

In the OPAT and HITH setting, one complex issue is the potential conflict between the choice of the most effective and/or narrow-spectrum agent, and the need for convenience in dosing and administration.^19^^,^^20^ Antimicrobial selection should be based on appropriate prescribing principles, but this could be limited by the logistical considerations of drug delivery via an OPAT service. In this study, this was illustrated in both the use of piperacillin/tazobactam (where a single drug may provide broad-spectrum therapy) and ceftriaxone (where a single daily dose is convenient). While there were circumstances where these antimicrobials were appropriate, in many cases the rationale for the use of such broad-spectrum agents was unknown. Similarly, other once-daily agents such as teicoplanin, daptomycin and ertapenem have the potential to be used for OPAT indications where they have unnecessarily broader-spectrum activity than required.

It is possible that in the OPAT setting the clinician’s ability to consult with other physicians may be limited, resulting in increased inappropriate prescribing practices. Ceftriaxone was prescribed in 10% of cases in this study, many of which were RTIs. The Australian ‘Therapeutic Guidelines: Antibiotic’ recommends utilizing ceftriaxone only in cases of severe pneumonia.^21^ Aside from the use of ceftriaxone for residents in skilled nursing facilities and residential aged care settings, it is unclear whether other patients fulfilling criteria for severe pneumonia would be treated in the OPAT setting.

The HITH-NAPS pilot also identified cases of prolonged parenteral therapy, for example, for treatment of cellulitis. The fact that the patients are well enough to be at home implies that most would be eating and drinking comfortably and might already be able to switch to oral antibiotic therapy. This may be an area for further work to minimize days of unnecessary IV access.^22^ Furthermore, as new data emerge that support earlier use of oral therapies (e.g. for bone and joint or soft tissue infection), practice within the OPAT and HITH setting should evolve to encompass earlier IV-to-oral switch where appropriate.^23–25^

OPAT and HITH should operate within the context of an AMS programme, with a microbiologist or infectious diseases physician involved in the initial design of antimicrobial protocols and available to provide advice to help guide ongoing patient care as needed.^19^ Several studies have found that assessment of referred patients by an infection specialist prior to OPAT resulted in reduced use of IV therapy, improved clinical care and substantial cost savings.^26–28^ An OPAT AMS checklist has been proposed as a means of providing guidance to OPAT services about stewardship.^20^ In regional settings, local access to infectious diseases and microbiology support is often limited. Antimicrobial prescribing support may require technologies such as telehealth with infectious diseases physicians and microbiologist teams from larger health centres or other remote access solutions.^29^

Nevertheless, we acknowledge that our study relied on local sites to provide data and self-report prescribing practices on a voluntary basis, and therefore the data do not encompass all Australian HITH and OPAT services. The audit was not able to correlate antimicrobial utilization with microbiology data or local microbial epidemiology that might have guided antimicrobial use, and variability in assessing appropriateness has been reported in other settings.^30^ As the data presented are from a 4 month time period, it is possible that conclusions regarding prescribing practices may not be widely generalizable. Encouragingly, this study demonstrates that HITH services are receptive to being benchmarked against other services with respect to antimicrobial prescribing practices, with a relatively high number of prescriptions assessed as appropriate.

### Conclusions

This study demonstrates that an NAPS for the OPAT setting is both feasible and valuable. Given the complexity of antimicrobial choice this study suggests that antimicrobial prescribing in the OPAT and HITH setting should operate under the guidance of an AMS programme. By providing valuable real-time data, HITH-NAPS has the capacity to encourage quality improvement to optimize patient outcomes, limit the development of antimicrobial resistance, facilitate benchmarking between health services and potentially reduce healthcare costs.

## Supplementary Material

dlaa058_Supplementary_Data
