# Supplementary material for: Measuring antimicrobial prescribing quality in outpatient parenteral antimicrobial therapy (OPAT) services: development and evaluation of a dedicated national antimicrobial prescribing survey
Source: JAC Antimicrob Resist. 2020 Aug 6;2(3):dlaa058. doi: 10.1093/jacamr/dlaa058 (PMC8210186; doi:10.1093/jacamr/dlaa058)

**Supplementary data**

**Appendix S1**. HITH NAPS data collection form


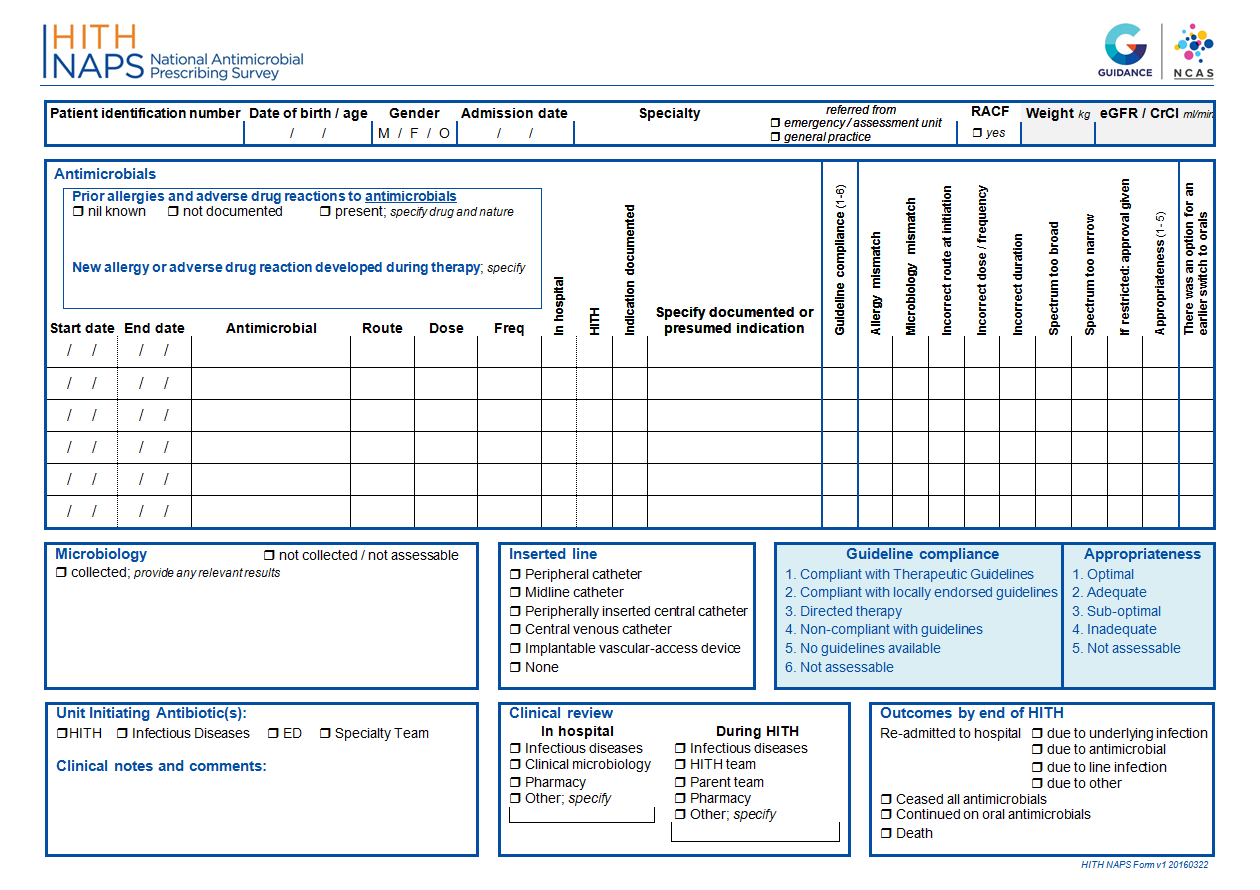


**Appendix S2**. NCAPS Appropriateness Evaluation Guide


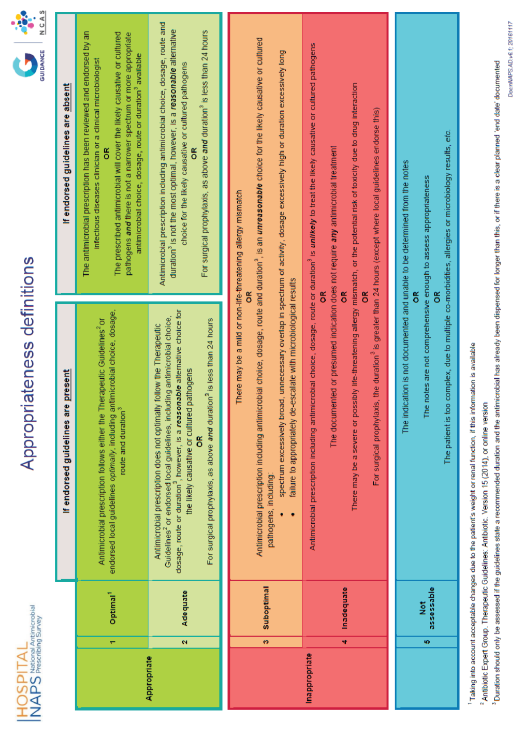

Supplement: dlaa058_Supplementary_Data [file dlaa058_supplementary_data.docx]
